# Prevalence and predictors of breastfeeding in the MINA-Brazil cohort

**DOI:** 10.11606/s1518-8787.2023057005563

**Published:** 2024-02-01

**Authors:** Paola S. Mosquera, Bárbara H. Lourenço, Alicia Matijasevich, Marcia C. Castro, Marly A. Cardoso

**Affiliations:** I Universidade de São Paulo Faculdade de Saúde Pública Departamento de Nutrição São Paulo Brasil Universidade de São Paulo. Faculdade de Saúde Pública. Departamento de Nutrição. São Paulo, Brasil; II Universidade de São Paulo Faculdade de Medicina Departamento de Medicina Preventiva São Paulo Brasil Universidade de São Paulo. Faculdade de Medicina. Departamento de Medicina Preventiva. São Paulo, Brasil; III Harvard T.H. Chan School of Public Health Department of Global Health and Population Boston MA Estados Unidos Harvard T.H. Chan School of Public Health. Department of Global Health and Population. Boston, MA, Estados Unidos.

**Keywords:** Breastfeeding, Survival Analysis, Risk Factors, Cohort Studies

## Abstract

**OBJECTIVE:**

To describe the prevalence and factors associated with exclusive (EBF) and continued breastfeeding (BF) practices among Amazonian children.

**METHODS:**

Data from 1,143 mother-child pairs recorded on the Maternal and Child Health and Nutrition in Acre (MINA-Brazil) birth cohort were used. Information on EBF and BF was collected after childbirth (July 2015–June 2016) and during the follow-up visits at 1 and 6 months postpartum, 1, 2, and 5 years of age. For longitudinal analysis, the outcomes were EBF and BF duration. Probability of breastfeeding practices were estimated by Kaplan-Meier survival analysis. Associations between baseline predictors variables and outcomes among children born at term were assessed by extended Cox regression models.

**RESULTS:**

EBF frequencies (95% confidence interval [95%CI]) at 3 and 6 months of age were 33% (95%CI: 30.2–36.0) and 10.8% (95%CI: 8.9–12.9), respectively. Adjusted hazard ratio for predictors of early EBF cessation were: being a first-time mother = 1.47 (95%CI: 1.19–1.80), feeding newborns with prelacteals = 1.70 (95%CI: 1.23–2.36), pacifier use in the first week of life = 1.79 (95%CI: 1.44–2.23) or diarrhea in the first two weeks of life = 1.70 (95%CI: 1.15–2.52). Continued BF frequency was 67.9% (95%CI: 64.9–70.8), 29.3% (95%CI: 26.4–32.4), and 1.7% (95%CI: 0.9–2.8) at 1, 2 and 5 years of age, respectively. Adjusted hazard ratio for predictors of early BF cessation were: male sex = 1.23 (95%CI: 1.01–1.49), pacifier use in the first week of life = 4.66 (95%CI: 2.99–7.26), and EBF less than 3 months = 2.76 (95%CI: 1.64–4.66).

**CONCLUSIONS:**

EBF and continued BF duration among Amazonian children is considerably shorter than recommendations from the World Health Organization. Significant predictors of breastfeeding practices should be considered for evaluating local strategies to achieve optimal breastfeeding practices.

## INTRODUCTION

The World Health Organization (WHO)^1^ and the Brazilian Ministry of Health recommend exclusive breastfeeding (EBF) for up to 6 months of age and continued breastfeeding (BF) until 2 years or more along with adequate complementary feeding. Benefits^2^ for maternal, child and population health reinforce this guidance. Nevertheless, the 2013-2018 global rates of EBF under six months and BF up to two years were approximately 41% and 45%, respectively^3^.

Since 1981, after implementation of the National Breastfeeding Incentive Program, several successful measures adopted by Brazil to improve BF rates^4^ have positioned the country as a model for implementing breastfeeding policies. However, despite tangible progress and continuous efforts made in the last decades, results from the *Estudo Nacional de Alimentação e Nutrição Infantil* (ENANI – Brazilian National Study of Child Nutrition)^5^, conducted in 2019 with a probabilistic sample of children under 5 years of age, showed a 45.8% EBF prevalence among infants younger than 6 months, with lower prevalence in Northern (40%) and Northeastern (39%) Brazil. BF frequency among children aged 20–23 months was 35.5%, with higher rates observed in the Northeast (48%), South (43%), and North (39%) regions.

In light of the 2030 WHO goals for at least 70% of EBF among infants under 6 months and 60% of BF at 2 years of age^3^, the latest national figures have fallen short of the expected global goals. According to a geospatial analysis of EBF prevalence in low- and middle-income countries (LMICs) from 2000 to 2018, Brazil has a low probability of meeting the EBF collective goals^6^. Thus, the country must intensify its efforts to protect, promote, and support breastfeeding taking into consideration the rates of BF practices^4^ and their determinants^7^, which vary according to specific regions and contexts.

Given the current challenging scenario, local investigations on breastfeeding duration predictors, together with general data provided by nationwide surveys, are crucial to optimize public health efforts, especially among vulnerable populations. However, few studies have focused on factors affecting BF rates among Amazonian children^8–10^, who have the most unfavorable living conditions in Brazil^11^. We hypothesized that sociodemographic, obstetric, perinatal, and mother-child characteristics at early life would be associated with EBF and BF duration in an urban Amazonian setting. Here, we describe the prevalence and factors associated with breastfeeding practices among children from birth to five years of age in an Amazonian city.

## METHODS

### Study Design and Population

Our study population consisted of mother-child pairs enrolled in the MINA-Brazil (Maternal and Child Health and Nutrition in Acre), a population-based birth cohort study conducted in Cruzeiro do Sul, Acre State, Western Brazilian Amazon. With roughly 90,000 inhabitants, of which 72% live in the urban area, the municipality has only one maternity hospital. Despite not being certified as a Baby Friendly Hospital, the facility has rooming-in beds, assistance by the kangaroo method, a weekly course on the Shantala technique for babies from the first month of life, and a human milk bank^8^.

Between July 2015 and June 2016, all women who gave birth in the local maternity and agreed to participate in the study were interviewed up to 12 hours after delivery. Follow-up assessments were performed by phone interviews one month after delivery and by visits to healthcare units when the children were 6–8 months, 1, 2, and 5 years of age^12^. Only single live births without any contraindication for breastfeeding^13^ were eligible for the present analysis. Mother-child pairs who did not participate in any of the follow-up assessments were excluded as no data on EBF or BF duration were available. Written informed consent was obtained at enrollment from study participants or from caregivers in the case of teenage mothers. The ethical review board of the Faculdade de Saúde Pública da Universidade de São Paulo (# 872.613, 2014; # 2.358.129, 2017) approved all the research procedures.

### Data Collection and Procedures

At baseline, the following maternal and perinatal covariates were collected by face-to-face interviews or from maternity medical records^12^: maternal age at delivery (< 19 or ≥ 19 years), maternal schooling (≤ 9 or > 9 years), self-reported skin color (white or non-white: black, brown, indigenous, and yellow), mother living with a partner (yes or no), wealth index (below or above average, as estimated by principal component analysis based on household assets), parity (primiparous or multiparous), number of antenatal care visits (ANC, < 6 or ≥ 6 visits), smoking during pregnancy (yes or no), maternal body weight at delivery (g), gestational age at delivery (GA, in weeks), type of delivery (vaginal or cesarean), child's sex (male or female), birthweight (BW, g), breastfeeding in the first hour (yes or no), and prelacteal feeding (yes or no). GA at delivery was categorized as less than 37 weeks (yes or no) to define preterm birth. BW (g) was categorized as low BW (LBW < 2,500 g). Small for GA (SGA, BW for GA < 10^th^ percentile) was defined using the Intergrowth-21^st^ Project charts for newborn size according to gestational age and sex. Data on malaria during pregnancy (yes or no) were obtained from the Ministry of Health's electronic database, as described elsewhere^12^.

Maternal height (m) and pre-pregnancy weight (kg) were collected from the prenatal card. Pre-pregnancy body mass index (BMI) was categorized as underweight (< 18.5 kg/m^2^), normal weight (18.5–24.9 kg/m^2^), overweight (25.0–29.9 kg/m^2^), or obese (≥ 30.0 kg/m^2^) according to WHO specifications. For the current analysis, we further categorized the pre-pregnancy BMI as < 25 kg/m^2^ or ≥ 25 kg/m^2^. Maternal gestational weight gain (GWG) was estimated by the difference between weight at delivery and pre-pregnancy weight. Based on pre-pregnancy BMI categories, GWG was classified as insufficient, adequate, or excessive according to the Institute of Medicine 2009 guidelines.

At the 1-month follow-up interview, we obtained additional information on the occurrence of sore breast or cracked nipples in the puerperium (yes or no), pacifier use (yes or no) and child's age in days at which the pacifier was offered, and infant health conditions, such as diarrhea, fever, wheezing and dry cough (yes or no), and the corresponding child's age at such episodes^8^. Pacifier use was categorized as in the first week of life (yes or no). Occurrence of diarrhea, fever, wheezing, and dry cough were categorized in the first 15 days of life (yes or no).

In all follow-up interviews, we asked the mothers whether the child was being breastfed (yes or no) and, if not, the age of weaning. At 1- and 6-month interviews, we assessed the age of introduction of liquids, semi-solid and solid foods since birth, as well as bottle use in the first 6 months (yes or no).

Children fed breast milk without other foods or drinks, including water, except prescribed medicines, oral rehydration solutions, vitamins and minerals, as defined by the WHO, were considered exclusively breastfed. Outcomes of interest consisted of EBF continuous variables in the first 6 months of life and BF during the first 5 years of age (in days). Minimum confirmed EBF or BF duration was used for children without data on EBF or BF cessation due to missed follow-ups (EBF, n = 58; BF, n = 279). EBF was also categorized as < 3 and ≥ 3 months to be analyzed as a exposure of continued BF.

### Statistical Analysis

Maternal and child characteristics were described as absolute frequencies and proportions (%). Baseline characteristics of the participants were compared with those lost to follow-up by using the chi-square test. EBF prevalence at 3 and 6 months, and BF prevalence at 1, 2, and 5 years of age were estimated, with their respective 95% confidence intervals (95%CI). The median duration of EBF and BF, and their probability at any point in time up to 6 months and up to 5 years, respectively, were estimated using Kaplan-Meier survival analysis. Children who were exclusively breastfed at 6 months or continued to be breastfed at 5 years of age were censored cases for this analysis, as were those lost to follow-up. Children who interrupted EBF or BF within the study period were considered failures.

According to the Schoenfeld global test and visual inspection of Kaplan-Meier survival curves, the proportional hazards assumption for Cox models was not met; thus, extended Cox regression models with fixed and time-varying covariates were performed to estimate the associations between exposure variables and EBF interruption before six months and BF cessation before two years. This analysis excluded preterm infants due to the greater risk of feeding difficulties^13^. Results were expressed as crude and adjusted hazard ratios (HR_a_) with 95%CI. Statistical significance was set at p < 0.05. The selection of exposures followed conceptual hierarchical models of factors associated with EBF^14^ and BF^15^ at four levels of determination: distal (socio-economic and demographic factors), distal-intermediate (obstetric factors), intermediate-proximal (perinatal characteristics) and proximal (mother-child characteristics in early life). Distal estimates were adjusted for all variables at that level of determination; those associated with the outcome were retained for model adjustment at the subsequent levels. Statistical analyses were performed using Stata version 15.0 (StataCorp, College Station, TX, USA).

## RESULTS

The MINA-Brazil birth cohort study comprised 1,246 participants at baseline. After exclusion of 22 twins, one newborn with cleft palate, and one HIV-positive mother, a total of 1,222 mother-child pairs remained eligible for the present analysis. Of these, 138 mother-child pairs did not participate in either the 1- or 6-month follow-up assessments, and 79 did not participate in any of the follow-up visits conducted up to 5 years of age (six children died; of these, three lacked data for breastfeeding). Information on EBF and BF duration was therefore available for 1,084 (88.7% of those eligible) and 1,143 (93.5% of those eligible) participants, respectively. The women excluded from the analysis due to lack of EBF or BF information had similar sociodemographic characteristics to those included, except for schooling level (< 9 years of schooling: EBF, 34.1% *versus* 45.8%; BF, 34.3% *versus* 51.3%) and wealth index (below average: EBF, 48.9% *versus* 59.4%; BF, 48.7% *versus* 69.7%).

Table 1 summarizes the baseline characteristics of mother-child pairs. Among the participating women, 87.6% declared themselves to be non-white (78.4% brown, 3.4% black, 4.6% yellow, and 1.2% Indigenous). About half of the women were primiparous (46.5%) and had a vaginal delivery (53.5%). As for newborns, 7.7% were born preterm and 13.1% were fed prelacteals (87.8% formula, 11.5% glucose water, and one child was given parenteral nutrition), a practice more prevalent among preterm (50%) than term (10%) newborns (p < 0.01). In the postnatal period, 12.2% of the newborns were offered a pacifier in the first week of life.

**Table 1:** Participant characteristics at baseline in the MINA-Brazil birth cohort with breastfeeding status information up to 5 years of age (n = 1,143).

Mother's characteristics		Total of participants n = 1,143^a^	Child's characteristics		Total of participants n = 1,143^a^
		n (%)			n (%)
Maternal age at delivery (years)			Sex		
	< 19	207 (18.1)		Female	572 (50.1)
	≥ 19	936 (81.9)		Male	571 (49.9)
Maternal schooling (years)			Preterm birth		
	≤ 9	382 (34.4)		Yes (< 37 weeks)	88 (7.7)
	> 9	730 (65.6)		No (≥ 37 weeks)	1,055 (92.3)
Maternal self-reported skin color			Low birth weight		
	White	138 (12.4)		Yes (< 2,500 grams)	79 (6.9)
	Black, brown, indigenous, and yellow	975 (87.6)		No (≥ 2,500 grams)	1,063 (93.1)
Woman living with her partner			Small for gestational age		
	Yes	860 (77.3)		Yes	94 (8.2)
	No	253 (22.7)		No	1,048 (91.8)
Household wealth index			Breastfeeding within the first hour		
	Below average	558 (50.1)		Yes	956 (88.6)
	Above average	555 (49.9)		No	123 (11.4)
Parity			Prelacteal feeding		
	Primiparous	517 (46.5)		Yes	149 (13.1)
	Multiparous	596 (53.5)		No	994 (86.9)
Antenatal care visits			Pacifier use in the first week of life		
	< 6	256 (22.6)		Yes	128 (12.2)
	≥ 6	879 (77.4)		No	920 (87.8)
Smoking during pregnancy			Diarrhea in the first 15 days of life		
	Yes	51 (4.6)		Yes	32 (3.6)
	No	1,062 (95.4)		No	856 (96.4)
Malaria during pregnancy			Fever in the first 15 days of life		
	Yes	79 (6.9)		Yes	62 (7.0)
	No	1,064 (93.1)		No	826 (93.0)
Pre-pregnancy body mass index^b^			Wheezing in the first 15 days of life		
	<25 Kg/m^2^	678 (64.8)		Yes	153 (17.2)
	≥ 25 Kg/m^2^	369 (35.2)		No	735 (82.8)
Gestational weight gain^c^			Dry cough in the first 15 days of life		
	Not excessive	688 (65.8)		Yes	30 (3.4)
	Excessive	357 (34.2)		No	858 (96.6)
Type of delivery			Bottle use in the first 6 months		
	Vaginal	612 (53.5)		Yes	806 (83.4)
	Cesarean	531 (46.5)		No	161 (16.6)
Breast problems in the puerperium^d^				
	Yes	509 (57.4)			
	No	378 (42.6)			

a Totals may differ due to missing values.

b According to the World Health Organization standards (WHO, 1995).

c According to the Institute of Medicine Guidelines, 2009.

d Sore breast, cracked nipples or both.

**Figure 1:**
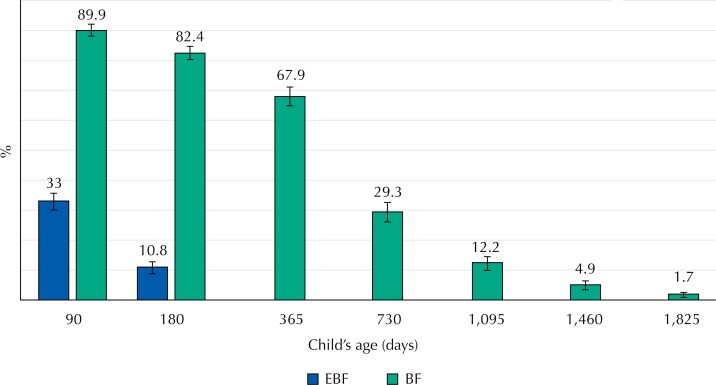
Prevalence (%) of exclusive breastfeeding (EBF) and continued breastfeeding (BF) among children over 5 years of age part of the MINA-Brazil birth cohort study. The bars represent 95% confidence intervals.

**Figure 2:**
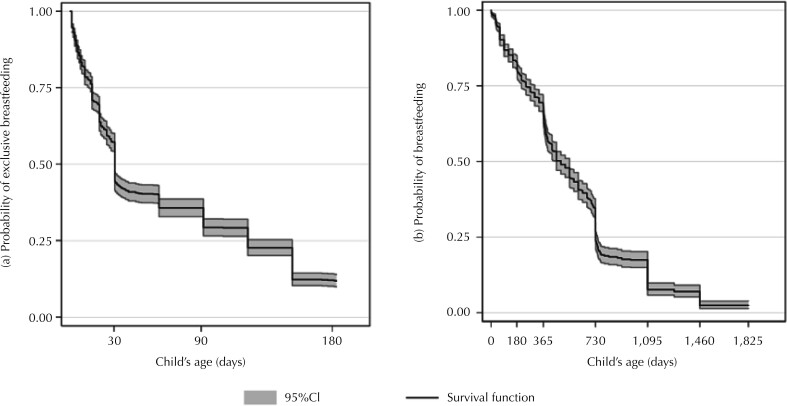
Kaplan-Meier survival curve for (a) exclusive breastfeeding up to 6 months and (b) breastfeeding up to 5 years of age among children in the MINA-Brazil birth cohort study.

EBF prevalence was 33% (95%CI: 30.2–36.0) at 3 months of age, and decreased to 10.8% (95%CI: 8.9–12.9) at 6 months. Although 67.9% (95%CI: 64.9–70.8) of the children were breastfed for up to 1 year of age, only 29.3% (95%CI: 26.4–32.4) continued to breastfeed for up to 2 years. At 5 years of age, 1.7% (95%CI: 0.9–2.8) of the children were still breastfed. The median duration of exclusive and continued breastfeeding was 30 days and 457 days, respectively (Figure 1). Figure 2 shows the Kaplan-Meier survival curves. Considering all children eligible for follow-up, the probability of infants being exclusive breastfed at 3 and 6 months of age was 29.3% and 11.8%, respectively. Median EBF duration was 30 days. The probability of children being breastfed at 1, 2, and 5 years of age was 65.5%, 34.5%, and 2.4%, respectively. Median BF duration was 488 days (16 months).

Table 2 summarizes the crude and adjusted estimates for factors affecting EBF duration up to 6 months of age among term infants (n = 1,003). The final model, time-dependent for parity and prelacteal feeding, showed no effect of ANC visits, breast problems, and newborn episodes of fever, wheezing and dry cough on EBF duration. However, children of primiparous women presented a 47% higher risk of early EBF cessation (HR_a_ = 1.47; 95%CI: 1.19–1.80) compared with babies of multiparous mothers. Similarly, newborns who were fed prelacteals (HR_a_ = 1.70; 95%CI: 1.23–2.36) or those who were offered a pacifier in the first week of life (HR_a_ = 1.79; 95%CI: 1.44–2.23) presented higher risks of early EBF cessation, when compared with their counterparts. Occurrence of diarrhea within the first 15 days of life was also associated with earlier EBF cessation (HR_a_ = 1.70; 95%CI: 1.15–2.52).

**Table 2:** Crude and adjusted extended Cox regression models for predictors of exclusive breastfeeding cessation before 6 months of age among term infants in the MINA-Brazil cohort.

Characteristics	Crude HR (95%CI)	Distal level	Distal intermediate level	Proximal intermediate level	Proximal level
		HRa (95%CI)	HRa (95%CI)	HRa (95%CI)	HRa (95%CI)
Maternal age at delivery < 19 years	1.29 (1.08–1.53)	0.98 (0.79–1.21)			
Maternal schooling ≤ 9 years	1.14 (0.99–1.32)	1.15 (0.98–1.35)			
Maternal non-white self-reported skin color	1.09 (0.87–1.35)	1.03 (0.82–1.28)			
Woman living with her partner	0.77 (0.65–0.90)	0.84 (0.71–1.00)			
Household Wealth Index below mean	1.12 (0.97–1.28)	1.07 (0.93–1.24)			
Primiparous mother^a^	1.63 (1.34–1.97)	1.62 (1.32–2.00)	1.73 (1.41–2.13)	1.72 (1.41–2.10)	1.47 (1.19–1.80)
Antenatal care visits < 6	1.21 (1.02–1.43)		1.34 (1.11–1.62)	1.30 (1.09–1.56)	1.17 (0.97–1.41)
Smoking during pregnancy	1.25 (0.90–1.72)		1.37 (0.96–1.96)		
Malaria during pregnancy	1.07 (0.83–1.38)		1.11 (0.84–1.46)		
Pre-pregnancy body mass index^b^ > 25 kg/m^2^	1.04 (0.90–1.20)		1.08 (0.93–1.27)		
Excessive gestational weight gain^c^	1.05 (0.90–1.21)		1.07 (0.92–1.25)		
Cesarean delivery	0.81 (0.70–0.93)			0.89 (0.76–1.03)	
Male baby	1.11 (0.97–1.27)			1.12 (0.97–1.29)	
Low birth weight	0.93 (0.61–1.41)			0.79 (0.49–1.28)	
Small for gestational age	1.08 (0.84–1.39)			1.08 (0.81–1.44)	
Breastfeeding within the first hour	1.33 (1.02–1.73)			1.32 (1.00–1.74)	
Prelacteal feeding^a^	1.35 (1.00–1.83)			1.48 (1.05–2.08)	1.70 (1.23–2.36)
Breast problems in the puerperium^d^	1.22 (1.04–1.41)				1.11 (0.94–1.30)
Pacifier use in the first week of life	1.70 (1.40–2.07)				1.79 (1.44–2.23)
Diarrhea in the first 15 days of life	1.55 (1.06–2.26)				1.70 (1.15–2.52)
Fever in the first 15 days of life	1.27 (0.96–1.68)				1.20 (0.98–1.66)
Wheezing in the first 15 days of life	1.25 (1.03–1.51)				1.25 (0.94–1.66)
Dry cough in the first 15 days of life	0.96 (0.64–1.44)				0.81 (0.53–1.24)

HR: hazard ratio; HR_a_: adjusted hazard ratio; 95%CI: 95% confidence interval.

Note: Complete case analysis (proximal model, n = 793). Totals differ due to missing values for covariates.

a Time-varying covariates.

b According to the World Health Organization standards.

c According to the Institute of Medicine Guidelines, 2009.

d Sore breast, cracked nipples or both.

Table 3 shows crude and adjusted estimates for factors affecting BF duration up to 2 years of age among children born at term (n = 1,055). The final model, time-dependent for pacifier use and EBF up to 3 months of age, showed that male children (HR_a_ = 1.23; 95%CI: 1.01–1.49), infants who were offered a pacifier within the first week of life (HR_a_ = 4.66; 95%CI: 2.99–7.26), and those exclusively breastfed less than 3 months (HR_a_ = 2.76; 95%CI: 1.64–4.66) had a higher risk of BF cessation before 2 years of age, when compared with the reference group.

**Table 3:** Crude and adjusted extended Cox regression models for predictors of breastfeeding cessation before 2 years of age among term infants in the MINA-Brazil cohort.

Characteristics	Crude	Distal level	Distal intermediate level	Proximal intermediate level	Proximal level
	HR (95% CI)	HRa (95%CI)	HRa (95%CI)	HRa (95%CI)	HRa (95%CI)
Maternal age at delivery < 19 years	1.13 (0.91–1.40)	1.12 (0.87–1.44)			
Maternal schooling ≤ 9 years	0.96 (0.80–1.14)	0.97 (0.80–1.18)			
Maternal non-white self-reported skin color	0.79 (0.63–1.00)	0.78 (0.62–0.99)	0.78 (0.62–1.00)	0.82 (0.64–1.04)	0.76 (0.57–1.03)
Woman living with her partner	0.79 (0.65–0.95)	0.79 (0.64–0.96)	0.76 (0.62–0.92)	0.79 (0.65–0.97)	0.87 (0.70–1.09)
Household Wealth Index below mean	0.88 (0.75–1.03)	0.87 (0.73–1.04)			
Primiparous mother	1.01 (0.86–1.19)	0.91 (0.75–1.10)			
Antenatal care visits < 6	0.91 (0.74–1.13)		0.90 (0.71–1.13)		
Smoking during pregnancy	1.09 (0.74–1.60)		1.03 (0.69–1.54)		
Malaria during pregnancy	0.97 (0.71–1.33)		1.01 (0.73–1.40)		
Pre-pregnancy body mass index^a^ > 25 kg/m^2^	0.94 (0.79–1.12)		0.92 (0.77–1.10)		
Excessive gestational weight gain^b^	0.99 (0.83–1.18)		1.00 (0.84–1.20)		
Cesarean delivery	0.91 (0.77–1.07)			0.90 (0.75–1.07)	
Male baby	1.29 (1.09–1.51)			1.29 (1.09–1.53)	1.23 (1.01–1.49)
Low birth weight	1.27 (0.81–1.99)			1.16 (0.69–1.92)	
Small for gestational age	1.12 (0.91–1.60)			1.08 (0.79–1.51)	
Breastfeeding within the first hour	1.06 (0.78–1.43)			1.12 (0.80–1.50)	
Prelacteal feeding^c^	1.30 (0.80–2.10)			1.40 (0.84–2.35)	
Breast problems^d^	1.10 (0.91–1.33)				1.03 (0.84–1.26)
Pacifier use in the first week of life^c^	4.70 (3.15–7.00)				4.66 (2.99–7.26)
Diarrhea in the first 15 days of life	1.10 (0.67–1.68)				1.03 (0.64–1.68)
Fever in the first 15 days of life	1.30 (0.92–1.83)				1.42 (1.00–2.02)
Wheezing in the first 15 days of life	1.01 (0.80–1.28)				0.91 (0.70–1.17)
Dry cough in the first 15 days of life	1.57 (0.96–2.55)				1.22 (0.70–2.12)
Exclusive breastfeeding < 3 months^c^	2.82 (1.92–4.13)				2.76 (1.64–4.66)
Bottle use while breastfeeding	1.55 (1.16–2.06)				1.28 (0.91–1.81)

HR: hazard ratio; HR_a_: adjusted hazard ratio; 95%CI: 95% confidence interval.

Note: Complete case analysis (proximal model n = 701). Totals differ due to missing values for covariates.

a According to the World Health Organization standards.

b According to the Institute of Medicine Guidelines, 2009.

c Time-varying covariates.

d Sore breast, cracked nipples or both.

## DISCUSSION

Our findings for this specific Amazonian region indicate that a small proportion of infants were exclusively breastfed up to 3 (33%) and 6 months (10.8%) of life. Continued BF frequencies were 67.9%, 29.3%, and 1.7% at 1, 2, and 5 years of age, respectively. Our results show that obstetric, perinatal and mother-child characteristics in early life determine the duration of breastfeeding practices among Amazonian children. Primiparity, prelacteal feeding, pacifier use, and episodes of diarrhea in the first two weeks of life were predictors of EBF interruption among term infants. Being male, pacifier use in the first week of life, and lower EBF duration (< 3 months) were predictors of BF cessation before 2 years of age.

EBF rates can be considered below the Brazilian nationwide estimates for children between 4 and 5 months of age (23.3%), according to ENANI in 2019^5^. The continued BF percentages found were higher than the national estimate at 1 year (52.1%), and similar (35.5%)^5^ at 2 years. However, all indicators were below the WHO recommendations.

In a previous analysis, we observed that children of multiparous women were exclusively breastfed for longer within the first month of life^8^. The present findings corroborate that parity remained associated with EBF duration during the first six months. Children of primiparous women presented a 47% higher risk of early EBF cessation. Although primiparity has already been negatively associated with exclusive breastfeeding in several studies^14^, this finding is not unanimous^7^. Underlying this relationship is the hypothesis that unlike first-time mothers, women with previous experience in breastfeeding probably have greater knowledge about infant care and feeding, resulting in greater confidence to breastfeed. In line with this assumption, high frequencies of continued BF have already been registered in Northern Brazil^4^, suggesting that most mothers undergo lactation and could be better prepared for EBF maintenance. A previous study showed that first-time mothers had more doubts regarding infant feeding and were discharged from the hospital later than multiparous women^16^. Considering that no previous BF experience or a disappointing one may negatively impact BF of the next child^17^, greater efforts should be made in supporting primiparous women to achieve a first positive BF experience.

Although few maternal-infant health conditions justify the temporary or permanent use of breast milk substitutes^13^, we found that 13% of the newborns were prelacteal fed and presented a 70% higher risk of early EBF cessation. Our estimate was lower than the prevalence of prelacteal feeding found in LMICs (33.9%) from 2010 to 2019^18^ and the prevalence of formula supplementation during hospital stay reported by a Canadian population-based birth cohort study (25.9%) conducted between 2009 and 2012^19^. However, it was similar to that found in a cohort study conducted in Rio Branco, Acre state, where 15% of the infants received formula supplementation before hospital discharge^10^. A recent retrospective cohort study including 85 LMICs has shown that prelacteal feeding was inversely associated with EBF in children under six months of age. Moreover, children were more likely to be fed formula if given prelacteal feeds^18^. Similarly, a meta-analysis of prospective studies observed a strong relationship between prelacteal feeding and EBF cessation^20^. Our finding points to the need for targeted interventions to educate health professionals on the harmful consequences from unnecessary use of breast milk substitutes and on BF management for supporting mothers from the early stages of pregnancy to initiate and establish EBF.

The protective effect of breastfeeding against infectious diseases is well documented in the literature^2^. A previous study on the MINA-Brazil cohort showed that children who were born to women that had gestational malaria and were breastfed for at least 12 months presented a decreased risk for malaria infection during the first 2 years of life^21^. Respiratory and gastrointestinal illnesses have been reported in BF infants from low-income populations, which are exposed to precarious environments and have limited access to healthcare^8,22^. The present study found that diarrhea within the first 15 days of life was associated with earlier EBF cessation. Some studies suggested that most mothers continue to breastfeed their children when they get sick^23^; however, teas for colic and gas relief are commonly offered from the first days of life^24^ compromising EBF until the recommended age.

Pacifier use in the first week of life incurred in a risk 1.79 and 4.66 times higher, respectively, for EBF and BF cessation before recommended, showing that its negative effect extends beyond early life^8^. Despite previous evidence relating the use of pacifier to shorter EBF^14^ and BF duration^15^, the literature is conflicting^25^. Recently, the WHO revised the Baby-Friendly Hospital Initiative's Ten Steps to Successful Breastfeeding, updating step 9 to counsel mothers on the use and risks of feeding bottles, teats and pacifiers instead of entirely prohibiting them for term infants, enabling families to make informed decisions about using or avoiding artificial nipples until breastfeeding is successfully established. Concerning their use, the WHO alerts that hygiene, oral formation, and identification of feeding cues are some aspects to be cautious about^13^. A Brazilian study suggested that reducing the prevalence of pacifier use could effectively improve EBF duration^26^.

As for the child's sex, earlier BF cessation was more frequent among boys, with similar findings being described among Brazilian^15,27^ and US Hispanic^28^ populations. Sociocultural norms and perceptions about higher nutritional needs among male than female children, together with traditional gender views^28^, may influence parents’ decisions regarding BF duration. Given the positive impacts of BF on maternal and child lives, however, local measures are needed to promote BF for all children and to change feeding behaviors that disadvantage boys.

Lastly, children who were exclusively breastfed for less than 3 months had a 2.7-fold greater risk of shorter BF duration. Some Brazilian^15,29^ and international^30^ research has pointed out the association between longer EBF and longer BF duration. Estimates from a cohort study of mother-child pairs conducted in Porto Alegre, Brazil, showed that the probability of BF maintenance up to 2 years of age or more was 0.5% and 0.1% greater for each extra day of preventing the infant's exposure to water or tea and other kinds of milk, respectively^29^. Environmental and motivational factors that predispose exclusive breastfeeding may favor continued breastfeeding in subsequent weeks^30^. Moreover, mothers who offer liquids or food in addition to breast milk may experience lower milk production due to reduced feedings and less nipple stimulation^15,30^.

Possibility of selection bias due to losses to follow-up could be a study limitation; but most of the sociodemographic characteristics did not differ according to retention, indicating reliable association measures. Self-reported infant morbidities and prelacteal feeding obtained from medical reports could have impacted estimates due to over-representation. Residual confounding is also possible due to unmeasured factors, such as the level of maternal motivation and support to breastfeed. Moreover, we did not investigate environmental and societal structural factors, which may influence the effects of individual factors on breastfeeding practices. In turn, its strengths include the longitudinal cohort design in the Amazonian region; data collection on infant feeding practices from the first month up to 5 years of age, minimizing recall bias; and definition of EBF duration using the recall since birth method, preventing misclassification.

## CONCLUSION

EBF and BF cessation in this study population occurred earlier than recommended. EBF interruption before six months of age was more likely to occur among children who were born to primiparous women, were exposed to prelacteal feds and pacifiers, or had diarrhea early in life. Male children and those who used pacifier or were exclusively breastfed for less than 3 months were more likely to fail continued BF up to 2 years of age. As most of the risk factors described are modifiable, our findings reinforce the need to strengthen efforts to support, promote, and protect breastfeeding.
