# Supplementary material for: Prevalence and predictors of breastfeeding in the MINA-Brazil cohort
Source: Rev Saude Publica. 2024 Feb 1;57(Suppl 2):2s. doi: 10.11606/s1518-8787.2023057005563 (PMC10897961; doi:10.11606/s1518-8787.2023057005563)
Supplement: Translation

# Translation: Prevalência e preditores do aleitamento materno na coorte MINA-Brasil

**Authors:** Paola S. Mosquera, Bárbara H. Lourenço, Alicia Matijasevich, Marcia C. Castro, Marly A. Cardoso

**Affiliations:** I São Paulo Brasil Universidade de São Paulo. Faculdade de Saúde Pública. Departamento de Nutrição. São Paulo, Brasil; II São Paulo Brasil Universidade de São Paulo. Faculdade de Medicina. Departamento de Medicina Preventiva. São Paulo, Brasil; III Boston MA Estados Unidos Harvard T.H. Chan School of Public Health. Department of Global Health and Population. Boston, MA, Estados Unidos.

**Keywords:** Aleitamento Materno, Análise de Sobrevida, Fatores de Risco, Estudos de Coortes

## Abstract

**OBJETIVO:**

Descrever a prevalência e os fatores associados às práticas de aleitamento materno exclusivo (AME) e continuado (AM) entre crianças amazônicas.

**MÉTODOS:**

Foram utilizados dados de 1.143 pares mãe-filho registrados na coorte de nascimento Materno-Infantil no Acre (MINA-Brasil). As informações sobre AME e AM foram coletadas após o parto (julho de 2015–junho de 2016) e durante as consultas de acompanhamento com 1 e 6 meses pós-parto e com 1, 2 e 5 anos de idade. A análise longitudinal considerou a duração do AME e AM como desfechos. A probabilidade das práticas de aleitamento materno foi estimada pela análise de sobrevida de Kaplan-Meier. As associações entre as variáveis preditoras basais e os desfechos entre crianças nascidas a termo foram avaliadas por modelos de regressão de Cox estendidos.

**RESULTADOS:**

As frequências de AME (intervalo de confiança de 95% [IC95%]) aos 3 e 6 meses de idade foram de 33% (IC95%: 30,2–36,0) e 10,8% (IC95%: 8,9–12,9), respectivamente. A razão de risco ajustada para preditores de interrupção precoce do AME foi: ser primípara = 1,47 (IC95%: 1,19–1,80), alimentar recém-nascidos com pré-lácteos = 1,70 (IC95%: 1,23–2,36), usar chupeta na primeira semana de vida = 1,79 (IC95%: 1,44–2,23) e apresentar diarreia nas duas primeiras semanas de vida = 1,70 (IC95%: 1,15–2,52). A frequência do AM continuado foi de 67,9% (IC95%: 64,9–70,8), 29,3% (IC95%: 26,4–32,4) e 1,7% (IC95%: 0,9–2,8) aos 1, 2 e 5 anos de idade, respectivamente. A razão de risco ajustada para preditores de cessação precoce do AM foi: sexo masculino = 1,23 (IC95%: 1,01–1,49), uso de chupeta na primeira semana de vida = 4,66 (IC95%: 2,99–7,26) e AME menor que 3 meses = 2,76 (IC95%: 1,64–4,66).

**CONCLUSÕES:**

A duração do AME e do AM continuado entre crianças amazônicas é consideravelmente menor do que as recomendações da Organização Mundial da Saúde. Preditores significativos das práticas de aleitamento materno devem ser considerados na avaliação das estratégias locais para alcançar práticas ideais de aleitamento materno.

## INTRODUÇÃO

A Organização Mundial da Saúde (OMS)^1^ e o Ministério da Saúde recomendam o aleitamento materno exclusivo (AME) até os 6 meses de idade e o aleitamento materno continuado (AM) até os 2 anos ou mais, além de alimentação complementar adequada. Os benefícios^2^ para a saúde materna, infantil e populacional reforçam essa orientação. No entanto, as taxas globais de AME até seis meses e AM até dois anos no período de 2013–2018 foram de aproximadamente 41% e 45%, respectivamente^3^.

Desde 1981, após a implementação do Programa Nacional de Incentivo ao Aleitamento Materno, várias medidas bem-sucedidas adotadas no Brasil para melhorar os indicadores de AM^4^ posicionaram o país como modelo de políticas públicas de aleitamento materno. Mas, apesar dos avanços tangíveis e dos esforços contínuos realizados nas últimas décadas, os resultados do Estudo Nacional de Alimentação e Nutrição Infantil (ENANI)^5^, realizado em 2019 com amostra probabilística de crianças menores de 5 anos, mostraram prevalência de 45,8% de AME entre lactentes menores de 6 meses, com menor prevalência nas regiões Norte (40%) e Nordeste (39%). A frequência de AM entre crianças de 20 a 23 meses foi de 35,5%, sendo observadas as maiores taxas nas regiões Nordeste (48%), Sul (43%) e Norte (39%).

Considerando as metas da OMS para 2030 de pelo menos 70% de AME entre crianças menores de 6 meses e 60% de AM aos 2 anos de idade^3^, as últimas estimativas nacionais ficaram aquém das metas globais esperadas. De acordo com uma análise geoespacial da prevalência do AME em países de baixa e média renda (PBMRs) de 2000 a 2018, o Brasil tem baixa probabilidade de atingir as metas coletivas de AME^6^. Assim, o país deve intensificar seus esforços para proteger, promover e apoiar o aleitamento materno, levando em consideração a frequência das práticas de AM^4^ e seus determinantes^7^, que variam de acordo com regiões e contextos específicos.

Diante do atual cenário desafiador, as investigações locais sobre preditores da duração do aleitamento materno, juntamente com os dados gerais fornecidos por inquéritos de abrangência nacional, são cruciais para otimizar os esforços de saúde pública, principalmente entre as populações vulneráveis. No entanto, poucos estudos têm focado nos fatores que afetam a frequência de AM entre as crianças amazônicas^8–10^, que possuem as condições de vida mais desfavoráveis do Brasil^11^. A nossa hipótese foi que características sociodemográficas, obstétricas, perinatais e materno-infantis no início da vida estariam associadas à duração do AME e AM em um cenário urbano amazônico. Aqui, descrevemos a prevalência e os fatores associados às práticas de aleitamento materno entre crianças do nascimento até os cinco anos de idade em uma cidade amazônica.

## MÉTODOS

### Concepção e População do Estudo

A nossa população de estudo foi composta por pares mãe-filho inscritos no MINA-Brasil (Materno-Infantil no Acre), um estudo de coorte de nascimentos de base populacional realizado em Cruzeiro do Sul, Estado do Acre, Amazônia Ocidental. Com cerca de 90.000 habitantes, dos quais 72% vivem na área urbana, o município possui apenas uma maternidade. Apesar de não ser certificada como Hospital Amigo da Criança, a instituição conta com alojamento conjunto, atendimento pelo método canguru, curso semanal sobre a técnica Shantala para bebês a partir do primeiro mês de vida e banco de leite humano^8^.

Entre julho de 2015 e junho de 2016, todas as mulheres que deram à luz na maternidade local e concordaram em participar da pesquisa foram entrevistadas até 12 horas após o parto. A avaliação de acompanhamento foi realizada por meio de entrevistas telefônicas com um mês após o parto e por visitas às unidades de saúde quando as crianças tinham de 6 a 8 meses, 1, 2 e 5 anos de idade^12^. Apenas recém-nascidos de nascimento único sem contraindicação para amamentar^13^ foram elegíveis para a presente análise. Foram excluídos os pares mãe-filho que não compareceram a nenhuma das avaliações de acompanhamento, pois não havia dados disponíveis sobre a duração do AME ou AM. No momento da inscrição, foi obtido o consentimento informado por escrito dos participantes do estudo ou dos responsáveis, no caso de mães adolescentes. Todos os procedimentos de pesquisa foram aprovados pelo Comitê de Ética em Pesquisa da Faculdade de Saúde Pública da Universidade de São Paulo(# 872.613, 2014; # 2.358.129, 2017).

### Coleta de Dados e Procedimentos

No início do estudo, as seguintes covariáveis maternas e perinatais foram coletadas por meio de entrevistas presenciais ou dos prontuários da maternidade^12^: idade materna no parto (< 19 ou ≥ 19 anos); escolaridade materna (≤ 9 ou > 9 anos); cor da pele autorreferida (branca ou não-branca: preta, parda, indígena e amarela); mãe vivendo com companheiro (sim ou não); índice de riqueza (abaixo ou acima da média, estimado pela análise de componentes principais com base nos bens do domicílio); paridade (primípara ou multípara); número de consultas pré-natais (PNT, < 6 ou ≥ 6 consultas); tabagismo durante a gestação (sim ou não); peso corporal materno no parto (g); idade gestacional no parto (IG, em semanas); tipo de parto (vaginal ou cesariana); sexo da criança (masculino ou feminino); peso ao nascer (PN, g); aleitamento materno na primeira hora (sim ou não); e alimentação pré-láctea (sim ou não). A IG no parto foi categorizada em menos de 37 semanas (sim ou não) para definir prematuridade. O PN (g) foi categorizado em baixo peso ao nascer (BPN < 2.500 g). Pequeno para IG (PIG, PN para IG < percentil 10) foi definido com base nos gráficos do Projeto Intergrowth-21st para o tamanho do recém-nascido de acordo com a idade gestacional e o sexo. Os dados sobre malária na gestação (sim ou não) foram obtidos na base de dados eletrônica do Ministério da Saúde, conforme descrito anteriormente^12^.

A altura materna (m) e o peso pré-gestacional (kg) foram coletados do cartão da gestante. O índice de massa corporal (IMC) pré-gestacional foi categorizado em baixo peso (< 18,5 kg/m^2^), peso normal (18,5–24,9 kg/m^2^), sobrepeso (25,0–29,9 kg/m^2^) ou obesidade (≥ 30,0 kg/m^2^), de acordo com as especificações da OMS. Para as análises atuais, categorizamos ainda o IMC pré-gestacional como < 25 kg/m^2^ ou ≥ 25 kg/m^2^. O ganho de peso gestacional (GPG) foi estimado pela diferença entre o peso no parto e o peso pré-gestacional. Com base nas categorias de IMC pré-gestacional, o GPG foi classificado como insuficiente, adequado ou excessivo, de acordo com as diretrizes de 2009 do *Institute of Medicine*.

Na entrevista de acompanhamento no primeiro mês de vida, obtivemos informações adicionais sobre a ocorrência de mama dolorida ou fissuras mamilares no puerpério (sim ou não); o uso de chupeta (sim ou não) e a idade da criança quando a chupeta foi oferecida pela primeira vez; as condições de saúde do lactente, como diarreia, febre, sibilância e tosse seca (sim ou não); e a idade da criança correspondente nesses episódios^8^. O uso de chupeta foi categorizado como na primeira semana de vida (sim ou não). A ocorrência de diarreia, febre, sibilância e tosse seca foram categorizadas nos primeiros 15 dias de vida (sim ou não).

Em todas as entrevistas de acompanhamento, perguntou-se às mães se a criança estava sendo amamentada (sim ou não) e, em caso negativo, a idade do desmame. Nas entrevistas de 1 e 6 meses, avaliou-se a idade de introdução de alimentos líquidos, semissólidos e sólidos desde o nascimento, bem como o uso de mamadeira nos primeiros 6 meses (sim ou não).

Crianças alimentadas com leite materno sem outros alimentos ou bebidas incluindo água, exceto medicamentos prescritos, soluções de reidratação oral, vitaminas e minerais, conforme definido pela OMS, foram consideradas amamentadas exclusivamente. Os desfechos de interesse consistiram nas variáveis contínuas de AME nos primeiros 6 meses de vida e de AM nos primeiros 5 anos de idade (em dias). Foi utilizada a duração mínima confirmada de AME ou AM para as crianças sem dados sobre a interrupção de AME ou AM devido às faltas aos acompanhamentos (AME, n = 58; AM, n = 279). O AME também foi categorizado como < 3 e ≥ 3 meses para ser analisado como uma exposição ao AM continuado.

### Análise Estatística

As características maternas e infantis foram descritas como frequências absolutas e proporções (%). O teste qui-quadrado foi utilizado para comparar as características das participantes na linha de base com aquelas perdidas no acompanhamento. Foram estimadas as prevalências de AME aos 3 e 6 meses, e AM com 1, 2 e 5 anos de idade, com os seus respectivos intervalos de confiança de 95% (IC95%). A análise de sobrevida de Kaplan-Meier foi utilizada para calcular a duração mediana do AME e do AM, e as suas probabilidades em qualquer momento até 6 meses e até 5 anos, respectivamente. As crianças que foram amamentadas exclusivamente aos 6 meses ou continuaram a ser amamentadas aos 5 anos de idade foram censuradas para esta análise, assim como aquelas com perda de acompanhamento. As crianças que interromperam o AME ou AM no período estudado foram consideradas falhas.

Como o pressuposto de riscos proporcionais para os modelos de Cox não foi atendida, de acordo com o teste global de Schoenfeld e a inspeção visual das curvas de sobrevida de Kaplan-Meier, realizamos modelos de regressão de Cox estendidos com covariáveis fixas e tempo-dependentes para estimar as associações entre as variáveis de exposição e a interrupção do AME e AM antes dos seis meses e antes dos dois anos de idade, respectivamente. Esta análise excluiu recém-nascidos prematuros devido ao maior risco de dificuldades para a amamentação^13^. Os resultados foram expressos como razões de risco (*hazard ratio,* em inglês) brutas e ajustadas (HR_a_) com IC95%. A significância estatística foi estabelecida em p < 0,05. A seleção de variáveis de exposição seguiu modelos hierárquicos conceituais de fatores associados ao AME^14^ e AM^15^ em quatro níveis de determinação: distal (fatores socioeconômicos e demográficos), distal-intermediário (fatores obstétricos), intermediário-proximal (características perinatais) e proximal (características mãe-filho no início da vida). As estimativas distais foram ajustadas para todas as variáveis nesse nível de determinação; aquelas associadas ao desfecho foram mantidas para ajuste do modelo nos níveis subsequentes. As análises estatísticas foram realizadas no programa Stata versão 15.0 (StataCorp, College Station, TX, EUA).

## RESULTADOS

O estudo de coorte de nascimentos MINA-Brasil incluiu 1.246 participantes na linha de base do estudo. Após a exclusão de 22 gêmeos, um recém-nascido com fissura palatina e uma mãe HIV positiva, um total de 1.222 pares mãe-filho permaneceram elegíveis para esta análise. Destes, 138 duplas mãe-filho não participaram das avaliações de acompanhamento de 1 ou 6 meses, e 79 não participaram de nenhuma das consultas de acompanhamento realizadas até os 5 anos de idade (seis crianças morreram; destas, três não tinham dados sobre amamentação). Assim, as informações sobre a duração do AME e AM estavam disponíveis para 1.084 (88,7% dos elegíveis) e 1.143 (93,5% dos elegíveis) participantes, respectivamente. As mulheres excluídas da análise por falta de informações sobre AME ou AM apresentaram características sociodemográficas semelhantes às incluídas, exceto escolaridade (< 9 anos de estudo: AME, 34,1% *versus* 45,8%; AM, 34,3% *versus* 51,3%) e índice de riqueza (abaixo da média: AME, 48,9% *versus* 59,4%; AM, 48,7% *versus* 69,7%).

A Tabela 1 resume as características basais dos pares mãe-filho. Entre as mulheres participantes, 87,6% declararam-se não-brancas (78,4% pardas, 3,4% negras, 4,6% amarelas e 1,2% indígenas). Cerca de metade das mulheres era primípara (46,5%) e teve parto vaginal (53,5%). Quanto aos recém-nascidos, 7,7% nasceram pré-termo e 13,1% receberam pré-lacteos (87,8% fórmula, 11,5% água glicosada e uma criança recebeu nutrição parenteral), prática mais prevalente entre os recém-nascidos pré-termo (50%) do que a termo (10%), (p < 0,01). No período pós-natal, 12,2% dos recém-nascidos receberam chupeta na primeira semana de vida.

**Table 1:** Características dos participantes na linha de base do estudo de coorte de nascimentos MINA-Brasil com informações sobre a situação do aleitamento materno até os 5 anos de idade (n = 1.143).

Características maternas		Total de participantes n = 1.143^a^	Características da criança		Total de participantes n = 1.143^a^
		n (%)			n (%)
Idade materna no parto (anos)			Sexo		
	< 19	207 (18,1)		Feminino	572 (50,1)
	≥ 19	936 (81,9)		Masculino	571 (49,9)
Escolaridade materna (anos)			Prematuridade		
	≤ 9	382 (34,4)		Sim (< 37 semanas)	88 (7,7)
	> 9	730 (65,6)		Não (≥ 37 semanas)	1.055 (92,3)
Cor da pele materna autorreferida			Baixo peso ao nascer		
	Branca	138 (12,4)		Sim (< 2.500 gramas)	79 (6,9)
	Preta, parda, indígena e amarela	975 (87,6)		Não (≥ 2.500 gramas)	1.063 (93,1)
Mulher vive com o companheiro			Pequeno para a idade gestacional		
	Sim	860 (77,3)		Sim	94 (8,2)
	Não	253 (22,7)		Não	1.048 (91,8)
Índice de Riqueza Familiar			Amamentação na primeira hora		
	Abaixo da média	558 (50,1)		Sim	956 (88,6)
	Acima da média	555 (49,9)		Não	123 (11,4)
Paridade			Alimentação pré-láctea		
	Primípara	517 (46,5)		Sim	149 (13,1)
	Multípara	596 (53,5)		Não	994 (86,9)
Consultas pré-natal			Uso de chupeta na primeira semana de vida		
	< 6	256 (22,6)		Sim	128 (12,2)
	≥ 6	879 (77,4)		Não	920 (87,8)
Tabagismo durante a gravidez			Diarreia nos primeiros 15 dias de vida		
	Sim	51 (4,6)		Sim	32 (3,6)
	Não	1.062 (95,4)		Não	856 (96,4)
Malária durante a gravidez			Febre nos primeiros 15 dias de vida		
	Sim	79 (6,9)		Sim	62 (7,0)
	Não	1.064 (93,1)		Não	826 (93,0)
Índice de massa corporal pré-gestacional^b^			Sibilância nos primeiros 15 dias de vida		
	<25 kg/m^2^	678 (64,8)		Sim	153 (17,2)
	≥ 25 kg/m^2^	369 (35,2)		Não	735 (82,8)
Ganho de peso gestacional^c^			Tosse seca nos primeiros 15 dias de vida		
	Não excessivo	688 (65,8)		Sim	30 (3,4)
	Excessivo	357 (34,2)		Não	858 (96,6)
Tipo de parto			Uso de mamadeira nos primeiros 6 meses		
	Vaginal	612 (53,5)		Sim	806 (83,4)
	Cesariana	531 (46,5)		Não	161 (16,6)
Problemas mamários no puerpério^d^				
	Sim	509 (57,4)			
	Não	378 (42,6)			

a Os totais podem diferir devido a valores em falta.

b De acordo com as normas da Organização Mundial da Saúde (OMS, 1995).

c De acordo com as Diretrizes do *Institute of Medicine*, 2009.

d Mama dolorida, mamilos rachados ou ambos.

A prevalência de AME foi de 33% (IC95%: 30,2–36,0) aos 3 meses de idade, e diminuiu para 10,8% (IC95%: 8,9–12,9) aos 6 meses. Embora 67,9% (IC95%: 64,9–70,8) dos lactentes tenham sido amamentados até 1 ano de idade, apenas 29,3% (IC95%: 26,4–32,4) continuaram sendo amamentados até 2 anos. Aos 5 anos de idade, 1,7% (IC95%: 0,9–2,8) das crianças ainda eram amamentadas. A duração mediana do AME e do AM continuado foi de 30 dias e 457 dias, respectivamente (Figura 1). A Figura 2 mostra as curvas de sobrevida de Kaplan-Meier. Considerando todas as crianças elegíveis ao acompanhamento, a probabilidade de os lactentes serem amamentados exclusivamente aos 3 e 6 meses de idade foi de 29,3% e 11,8%, respectivamente. A duração mediana do AME foi de 30 dias. A probabilidade de as crianças serem amamentadas com 1, 2 e 5 anos de idade foi de 65,5%, 34,5% e 2,4%, respectivamente. A duração mediana do AM foi de 488 dias (16 meses).

**Figura 1:** Prevalência (%) de aleitamento materno exclusivo (AME) e aleitamento materno continuado (AM) entre as crianças acima de 5 anos de idade, parte da coorte de nascimentos MINA-Brasil. As barras representam intervalos de confiança de 95%.

**Figura 2:** Curva de sobrevida de Kaplan-Meier para (a) aleitamento materno exclusivo até os 6 meses e (b) aleitamento materno até os 5 anos de idade entre as crianças do estudo de coorte de nascimentos MINA-Brasil.

A Tabela 2 resume as estimativas brutas e ajustadas para os fatores que afetam a duração do AME até os 6 meses de idade entre os recém-nascidos a termo (n = 1.003). O modelo de ajuste final tempo-dependente para paridade e alimentação pré-láctea não mostrou efeito de consultas PNT, problemas mamários e episódios neonatais de febre, sibilância e tosse seca na duração do AME. Entretanto, filhos de mulheres primíparas apresentaram um risco 47% maior de cessação precoce do AME (HR_a_ = 1,47; IC95%: 1,19–1,80) em comparação com bebês de mães multíparas. Da mesma forma, os recém-nascidos que receberam pré-lácteos (HR_a_ = 1,70; IC95%: 1,23–2,36) ou aqueles que receberam chupeta na primeira semana de vida (HR_a_ = 1,79; IC95%: 1,44–2,23) apresentaram maior risco de cessação precoce do AME, quando comparados aos seus pares. A ocorrência de diarreia nos primeiros 15 dias de vida também esteve associada à interrupção precoce do AME (HR_a_ = 1,70; IC95%: 1,15–2,52).

**Table 2:** Modelos brutos e ajustados de regressão de Cox estendidos para preditores de interrupção do aleitamento materno exclusivo antes dos 6 meses de idade entre os recém-nascidos a termo na coorte MINA-Brasil.

Características	Bruto	Nível distal	Nível distal-intermediário	Nível intermediário-proximal	Nível proximal
	HR (IC95%)	HRa (IC95%)	HRa (IC95%)	HRa (IC95%)	HRa (IC95%)
Idade materna no parto < 19 anos	1,29 (1,08–1,53)	0,98 (0,79–1,21)			
Escolaridade materna ≤ 9 anos	1,14 (0,99–1,32)	1,15 (0,98–1,35)			
Cor da pele materna autorreferida não-branca	1,09 (0,87–1,35)	1,03 (0,82–1,28)			
Mulher vive com o companheiro	0,77 (0,65–0,90)	0,84 (0,71–1,00)			
Índice de Riqueza Familiar abaixo da média	1,12 (0,97–1,28)	1,07 (0,93–1,24)			
Mãe primípara^a^	1,63 (1,34–1,97)	1,62 (1,32–2,00)	1,73 (1,41–2,13)	1,72 (1,41–2,10)	1,47 (1,19–1,80)
Consultas pré-natal < 6	1,21 (1,02–1,43)		1,34 (1,11–1,62)	1,30 (1,09–1,56)	1,17 (0,97–1,41)
Tabagismo durante a gravidez	1,25 (0,90–1,72)		1,37 (0,96–1,96)		
Malária durante a gravidez	1,07 (0,83–1,38)		1,11 (0,84–1,46)		
Índice de massa corporal pré-gestacional^b^ > 25 kg/m^2^	1,04 (0,90–1,20)		1,08 (0,93–1,27)		
Ganho de peso gestacional excessivo^c^	1,05 (0,90–1,21)		1,07 (0,92–1,25)		
Parto cesáreo	0,81 (0,70–0,93)			0,89 (0,76–1,03)	
Bebê do sexo masculino	1,11 (0,97–1,27)			1,12 (0,97–1,29)	
Baixo peso ao nascer	0,93 (0,61–1,41)			0,79 (0,49–1,28)	
Pequeno para a idade gestacional	1,08 (0,84–1,39)			1,08 (0,81–1,44)	
Amamentação na primeira hora	1,33 (1,02–1,73)			1,32 (1,00–1,74)	
Alimentação pré-láctea^a^	1,35 (1,00–1,83)			1,48 (1,05–2,08)	1,70 (1,23–2,36)
Problemas mamários no puerpério^d^	1,22 (1,04–1,41)				1,11 (0,94–1,30)
Uso de chupeta na primeira semana de vida	1,70 (1,40–2,07)				1,79 (1,44–2,23)
Diarreia nos primeiros 15 dias de vida	1,55 (1,06–2,26)				1,70 (1,15–2,52)
Febre nos primeiros 15 dias de vida	1,27 (0,96–1,68)				1,20 (0,98–1,66)
Sibilância nos primeiros 15 dias de vida	1,25 (1,03–1,51)				1,25 (0,94–1,66)
Tosse seca nos primeiros 15 dias de vida	0,96 (0,64–1,44)				0,81 (0,53–1,24)

HR: razão de risco; HR_a_: razão de risco ajustada; IC95%: Intervalo de confiança de 95%.

Nota: Análise de casos completos (modelo proximal, n = 793). Os totais diferem devido à falta de valores para as covariáveis.

a Covariáveis tempo-dependentes.

b De acordo com as normas da Organização Mundial da Saúde.

c De acordo com as Diretrizes do *Institute of Medicine*, 2009.

d Mama dolorida, mamilos rachados ou ambos.

A Tabela 3 mostra as estimativas brutas e ajustadas para os fatores que afetam a duração do AM até os 2 anos de idade entre os recém-nascidos a termo (n = 1.055). O modelo de ajuste final, tempo-dependente para uso de chupeta e AME até os 3 meses de idade, mostrou que crianças do sexo masculino (HR_a_= 1,23; IC95%: 1,01–1,49); lactentes aos quais foi oferecida chupeta na primeira semana de vida (HR_a_ = 4,66; IC95%: 2,99–7,26); e aqueles amamentados exclusivamente por menos de 3 meses (HR_a_ = 2,76; IC95%: 1,64–4,66) apresentaram maior risco de interrupçao do AM antes dos 2 anos de idade, quando comparados ao grupo de referência.

**Table 3:** Modelos brutos e ajustados de regressão de Cox estendidos para preditores de interrupção do aleitamento materno antes dos 2 anos de idade entre os recém-nascidos a termo na coorte MINA-Brasil.

Características	HR Bruto (IC95%)	Nível distal	Nível distal-intermediário	Nível intermediário-proximal	Nível proximal
		HRa (IC95%)	HRa (IC95%)	HRa (IC95%)	HRa (IC95%)
Idade materna no parto < 19 anos	1,13 (0,91–1,40)	1,12 (0,87–1,44)			
Escolaridade materna ≤ 9 anos	0,96 (0,80–1,14)	0,97 (0,80–1,18)			
Cor da pele materna autorreferida não-branca	0,79 (0,63–1,00)	0,78 (0,62–0,99)	0,78 (0,62–1,00)	0,82 (0,64–1,04)	0,76 (0,57–1,03)
Mulher vive com o companheiro	0,79 (0,65–0,95)	0,79 (0,64–0,96)	0,76 (0,62–0,92)	0,79 (0,65–0,97)	0,87 (0,70–1,09)
Índice de Riqueza Familiar abaixo da média	0,88 (0,75–1,03)	0,87 (0,73–1,04)			
Mãe primípara	1,01 (0,86–1,19)	0,91 (0,75–1,10)			
Consultas pré-natal < 6	0,91 (0,74–1,13)		0,90 (0,71–1,13)		
Tabagismo durante a gravidez	1,09 (0,74–1,60)		1,03 (0,69–1,54)		
Malária durante a gravidez	0,97 (0,71–1,33)		1,01 (0,73–1,40)		
Índice de massa corporal pré-gestacional^a^ > 25 kg/m^2^	0,94 (0,79–1,12)		0,92 (0,77–1,10)		
Ganho de peso gestacional excessivo^b^	0,99 (0,83–1,18)		1,00 (0,84–1,20)		
Parto cesáreo	0,91 (0,77–1,07)			0,90 (0,75–1,07)	
Bebê do sexo masculino	1,29 (1,09–1,51)			1,29 (1,09–1,53)	1,23 (1,01–1,49)
Baixo peso ao nascer	1,27 (0,81–1,99)			1,16 (0,69–1,92)	
Pequeno para a idade gestacional	1,12 (0,91–1,60)			1,08 (0,79–1,51)	
Amamentação na primeira hora	1,06 (0,78–1,43)			1,12 (0,80–1,50)	
Alimentação pré-láctea^c^	1,30 (0,80–2,10)			1,40 (0,84–2,35)	
Problemas mamários^d^	1,10 (0,91–1,33)				1,03 (0,84–1,26)
Uso de chupeta na primeira semana de vida^c^	4,70 (3,15–7,00)				4,66 (2,99–7,26)
Diarreia nos primeiros 15 dias de vida	1,10 (0,67–1,68)				1,03 (0,64–1,68)
Febre nos primeiros 15 dias de vida	1,30 (0,92–1,83)				1,42 (1,00–2,02)
Sibilância nos primeiros 15 dias de vida	1,01 (0,80–1,28)				0,91 (0,70–1,17)
Tosse seca nos primeiros 15 dias de vida	1,57 (0,96–2,55)				1,22 (0,70–2,12)
Aleitamento materno exclusivo < 3 meses^c^	2,82 (1,92–4,13)				2,76 (1,64–4,66)
Uso de mamadeira durante a amamentação	1,55 (1,16–2,06)				1,28 (0,91–1,81)

HR: razão de risco; HR_a_: razão de risco ajustada; IC95%: Intervalo de confiança de 95%.

Nota: Análise de casos completos (modelo proximal n = 701). Os totais diferem devido à falta de valores para as covariáveis.

a De acordo com as normas da Organização Mundial da Saúde.

b De acordo com as Diretrizes do *Institute of Medicine*, 2009.

c Covariáveis tempo-dependentes.

d Mama dolorida, mamilos rachados ou ambos.

## DISCUSSÃO

Nossos achados para essa região amazônica específica indicam que uma pequena proporção de crianças foi amamentada exclusivamente até os 3 (33%) e 6 meses (10,8%) de vida. As frequências de AM continuado foram de 67,9%, 29,3% e 1,7% aos 1, 2 e 5 anos de idade, respectivamente. Nossos resultados mostram que características obstétricas, perinatais e materno-infantis no início da vida determinam a duração das práticas de aleitamento materno entre as crianças amazônicas. Primiparidade, alimentação pré-láctea, uso de chupeta e episódios de diarreia nas duas primeiras semanas de vida foram preditores da interrupção do AME entre os recém-nascidos a termo. O fato de ser do sexo masculino, uso de chupeta na primeira semana de vida e o tempo menor de AME (< 3 meses) foram preditores da cessação do AM antes dos 2 anos de idade.

As frequências de AME podem ser consideradas abaixo das estimativas nacionais para crianças de 4 a 5 meses de idade (23,3%), segundo o ENANI em 2019^5^. Os percentuais de AM continuado encontrados foram superiores à estimativa nacional para 1 ano (52,1%) e semelhantes (35,5%)^5^ aos 2 anos. No entanto, todos os indicadores ficaram abaixo das recomendações da OMS.

Em análise anterior, observamos que filhos de mulheres multíparas foram amamentados exclusivamente por mais tempo no primeiro mês de vida^8^. Os nossos achados corroboram que a paridade permaneceu associada à duração do AME nos primeiros seis meses. Os filhos de mulheres primíparas apresentaram um risco 47% maior de cessação precoce do AME. Embora a primiparidade já tenha sido negativamente associada ao aleitamento materno exclusivo em vários estudos^14^, esse achado não é unânime^7^. Subjacente a essa relação está a hipótese de que, diferentemente das mães primíparas, as mulheres com experiência prévia em aleitamento materno provavelmente têm maior conhecimento sobre os cuidados e a alimentação infantil, resultando em maior confiança para amamentar. Seguindo essa premissa, altas frequências de AM continuado já foram registradas na região Norte do Brasil^4^, sugerindo que a maioria das mães passa pela lactação e poderia estar mais confiante para a manutenção do AME. Um estudo anterior mostrou que as mães de primeira viagem tinham mais dúvidas em relação à alimentação infantil e recebiam alta hospitalar mais tardiamente do que mulheres multíparas^16^. Considerando que a ausência de experiência prévia de AM ou uma experiência decepcionante pode impactar negativamente o AM do próximo filho^17^, devem ser empreendidos maiores esforços de apoio às mulheres primíparas, para que elas consigam ter uma primeira experiência positiva de AM.

Embora poucas condições de saúde materno-infantil justifiquem o uso temporário ou permanente de substitutos do leite materno^13^, observou-se que 13% dos recém-nascidos foram alimentados com pré-lácteos e apresentaram um risco 70% maior de interrupção precoce do AME. A nossa estimativa foi inferior à prevalência de alimentação pré-láctea encontrada em PBMRs (33,9%) de 2010 a 2019^18^, e à prevalência de suplementação com fórmula durante a internação hospitalar relatada em um estudo canadense de coorte de nascimentos de base populacional (25,9%) realizado entre 2009 e 2012^19^. No entanto, foi semelhante ao encontrado em um estudo de coorte realizado em Rio Branco, Acre, onde 15% dos lactentes receberam suplementação com fórmula antes da alta hospitalar^10^. Um recente estudo de coorte retrospectivo com 85 PBMRs mostrou que a alimentação pré-láctea foi inversamente associada ao AME em crianças menores de seis meses de idade. Além disso, as crianças eram mais propensas a receber fórmula se recebessem alimentação pré-láctea^18^. Da mesma forma, uma metanálise de estudos prospectivos observou uma forte relação entre alimentação pré-láctea e cessação do AME^20^. O nosso achado aponta para a necessidade de intervenções direcionadas à capacitação dos profissionais de saúde acerca das consequências nocivas do uso desnecessário de substitutos do leite materno, e acerca do manejo do AM para apoiar as mães desde os primeiros estágios da gestação a iniciar e estabelecer o AME.

O efeito protetor do aleitamento materno contra doenças infecciosas está bem documentado na literatura^2^. Um estudo anterior com dados da coorte MINA-Brasil mostrou que as crianças nascidas de mulheres com malária gestacional, amamentadas por pelo menos 12 meses, apresentaram risco reduzido de infecção por malária nos primeiros 2 anos de vida^21^. Doenças respiratórias e gastrointestinais têm sido relatadas em lactentes em AM em populações de baixa renda, expostos a ambientes precários e com acesso limitado aos cuidados de saúde^8,22^. O presente estudo verificou que a diarreia nos primeiros 15 dias de vida associou-se à interrupção mais precoce do AME. Alguns estudos sugerem que muitas mães continuam amamentando seus filhos quando estes adoecem^23^; no entanto, o uso de chás para alívio de cólicas e gases comumente oferecidos nos primeiros dias de vida^24^ podem comprometer o AME até a idade recomendada.

O uso de chupeta na primeira semana de vida implicou em um risco 1,79 e 4,66 vezes maior, respectivamente, para cessação do AME e AM antes do recomendado, mostrando que o seu efeito negativo se estende para além do início da vida^8^. Apesar de evidências prévias relacionando o uso de chupeta a um menor tempo de AME^14^ e AM^15^, a literatura é conflitante^25^. Recentemente, a OMS revisou os Dez Passos para o Sucesso do Aleitamento Materno da Iniciativa Hospital Amigo da Criança; o passo 9 foi revisado para aconselhar as mães sobre o uso e os riscos de mamadeiras, bicos e chupetas, em vez de proibi-los totalmente para os bebês a termo, permitindo que as famílias tomem decisões conscientes sobre usar ou evitar mamilos artificiais até que a amamentação seja estabelecida com sucesso. Em relação ao seu uso, a OMS alerta que a higiene, a formação oral e a identificação de sinais para a alimentação são alguns aspectos a serem considerados com precaução^13^. Um estudo brasileiro sugeriu que a redução da prevalência do uso de chupeta poderia melhorar a duração do AME^26^.

Quanto ao sexo da criança, a cessação mais precoce do AM foi mais frequente em meninos, com achados semelhantes sendo descritos em populações brasileiras^15,27^ e hispânicas norte-americanas^28^. As normas socioculturais e as percepções sobre maiores necessidades nutricionais em crianças do sexo masculino do que feminino, juntamente com visões tradicionais de gênero^28^, podem influenciar as decisões dos pais em relação à duração do AM. No entanto, diante dos impactos positivos do AM na vida materna e infantil, são necessárias medidas locais para promover o AM para todas as crianças e mudar comportamentos alimentares que prejudicam os meninos.

Por fim, as crianças que foram amamentadas exclusivamente por menos de 3 meses apresentaram risco 2,7 vezes maior de menor duração do AM. Algumas pesquisas nacionais^15,29^ e internacionais^30^ têm apontado a associação entre maior duração do AME e maior duração do AM. Estimativas de um estudo de coorte de pares mãe-filho realizado em Porto Alegre, Brasil, mostraram que a probabilidade de manutenção do AM até os 2 anos de idade ou mais foi 0,5% e 0,1% maior para cada dia a mais de prevenção da exposição do lactente à água, chá e outros tipos de leite, respectivamente^29^. Fatores ambientais e motivacionais que predispõem ao aleitamento materno exclusivo podem favorecer a continuidade do aleitamento materno nas semanas subsequentes^30^. Além disso, as mães que oferecem líquidos ou alimentos além do leite materno podem apresentar menor produção de leite devido à redução da amamentação e à menor estimulação mamilar^15,30^.

A possibilidade de viés de seleção devido às perdas de acompanhamento poderia ser uma limitação do estudo; porém, a maioria das características sociodemográficas não diferiu de acordo com a retenção, indicando medidas de associação confiáveis. Morbidades infantis autorreferidas e alimentação pré-láctea obtidas de laudos médicos podem ter impactado as estimativas devido à super-representação. O confundimento residual também é possível devido a fatores não mensurados, como o nível de motivação materna e apoio para amamentar. Além disso, não foram investigados fatores ambientais e estruturais da sociedade, que podem influenciar os efeitos de fatores individuais sobre as práticas de aleitamento materno. Por sua vez, os seus pontos fortes incluem a concepção do estudo de coorte longitudinal na região amazônica; coleta de dados sobre práticas alimentares infantis do primeiro mês até os 5 anos de idade, minimizando o viés de memória; e definição da duração do AME pelo método recordatório desde o nascimento, evitando erros de classificação.

## CONCLUSÃO

A interrupção do AME e AM na população estudada ocorreu antes do recomendado. A interrupção do AME antes dos seis meses de idade foi mais provável entre crianças nascidas de mulheres primíparas, expostas a alimentos pré-lácteos e chupetas, ou que tiveram diarreia no início da vida. Crianças do sexo masculino e aquelas que usavam chupeta ou foram amamentadas exclusivamente por menos de 3 meses apresentaram maior probabilidade de não continuarem a amamentar até os 2 anos de idade. Como a maioria dos fatores de risco descritos são modificáveis, os nossos achados reforçam a necessidade de fortalecer os esforços para apoiar, promover e proteger o aleitamento materno.
